# Strontium Ion Removal From Artificial Seawater Using a Combination of Adsorption With Biochar and Precipitation by Blowing CO_2_ Nanobubble With Neutralization

**DOI:** 10.3389/fbioe.2022.819407

**Published:** 2022-02-10

**Authors:** Yixuan Guo, Nguyen Thi Hong Nhung, Xiang Dai, Chunlin He, Youbin Wang, Yuezhou Wei, Toyohisa Fujita

**Affiliations:** ^1^ School of Resources, Environment and Materials, Guangxi University, Nanning, China; ^2^ School of Nuclear Science and Technology, University of South China, Hengyang, China; ^3^ School of Chemistry and Chemical Engineering, Guangxi University, Nanning, China

**Keywords:** Biochar, Adsorption, strontium radionuclide, Seawater, CO_2_ nanobubbles

## Abstract

While enjoying the convenience of nuclear energy development, the environmental contamination by radionuclide leakage is of significant concern. Because of its cost-effectiveness and environmental friendliness, biochar has attracted a lot of attention in the field of radioactive water treatment. Herein, a novel teak peel modified biochar (labeled as PMBN3) was prepared and applied to remove strontium from artificial seawater. The characterisation of the prepared PMBN3 showed it contains numerous oxygen-containing functional groups (i.e. carboxyl and hydroxyl groups), laminar morphology, mesoporous structure, large specific surface area. PMBN3 exhibited great advantages in Sr(II) adsorption, such as rapid adsorption kinetics (<1 h for equilibrium) and superior reusability. The adsorption of strontium by biochar is consistent with pseudo-second order and internal diffusion kinetic models. Among the four types of adsorption isotherms, the Freundlich isotherm showed the best fit with R_2_ > 0.98. The calculated thermodynamic parameters indicate that strontium adsorption on biochar occurs exothermically and spontaneously. Furthermore, for efficient removal of Sr(II), CO_2_ nanobubbles were blown into artificial seawater to precipitate the interfering metal ions, and followed by the adsorption of PMBN3 towards residual metal ions with the removal rate of Sr(II) over 99.7%. Finally, mechanistic studies have shown that the strontium adsorption process by PMBN3 is a multiple adsorption mechanism consisting of ion exchange between H^+^ (from -OH and -COOH) and Sr(II), and weak intermolecular forces between Sr(II) and the PMBN3 adsorbent. This study creatively combines chemisorption and nanobubble precipitation for strontium removal, which provides great reference value and guidance for environmental remediation.

**GRAPHICAL ABSTRACT:**
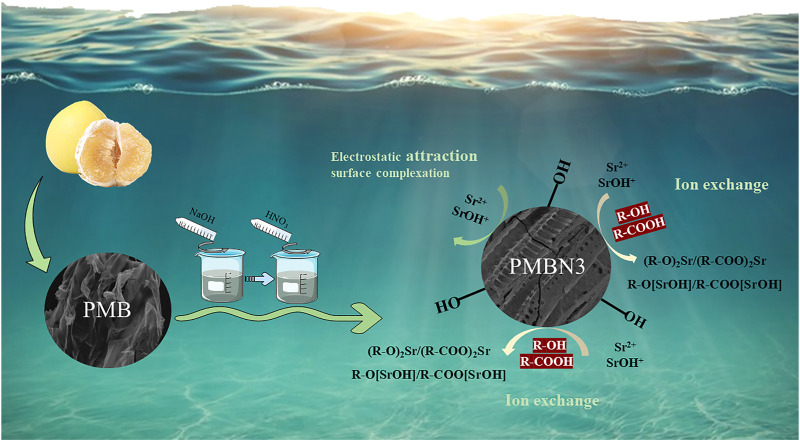


## 1 Introduction

In april 2021, the Japanese government decided to purify nuclear wastewater and discharge it into the sea has sparked widespread throughout the world. The Fukushima Daiichi Nuclear Disaster is widely known for releasing a variety of radionuclides including Cs-137/134, Sr-90, and H-3 into the atmosphere and oceans (Kirishima et al., 2014; Hori et al., 2018; Kato et al., 2019), which dramatically contaminated the ecosystem and posed a significant threat to human life. One of the most important radionuclides, Sr-90, has excellent water solubility and is easily deposited in bones after ingestion causing bone cancer, anemia, leukemia, and other diseases (Shin et al., 2021a). It is also regarded as one of the most serious radioactive contaminants on nuclear power plant sites and the damage of organisms in surrounding forests and oceans (Kirishima et al., 2014). Therefore, the removal of Sr-90 is of great significance for decrease of human health effects and ecological environmental remediation.

Following the Fukushima Daiichi Nuclear Disaster, several combination methods including adsorption (Awual et al., 2014), extraction (Tajima et al., 2019), membrane separation (Ding et al., 2019), and precipitation (Kosaka et al., 2012) were used to remove radioactive cesium and get good grades. After the Fukushima Daiichi Nuclear Disaster in Japan in 2011, various combination processes were used to treat radioactive cesium ions. However, Sr-90 interacts with alkali and alkaline earth metals and anions in diverse water bodies to change from free cations to complexes or colloids (Kirishima et al., 2014), making these combination processes incapable of completely removing radioactive strontium ions from the aqueous phase.

Many methods for removing Sr from aqueous phases have been developed in recent years, including biological methods (Chen and Wang., 2012), chemical precipitation (Su et al., 2020), solvent extraction, membrane separation (Cai et al., 2020), and adsorption (Huo et al., 2021). For the adsorption study, adsorption mechanism and capacity were described and novel adsorbents were prepared, however, Sr removal percentage discussion was low. Among them, the precipitation as Strontianite (SrCO_3_) has attracted great attention to remove Sr, for example 98% Sr removal (Su et al., 2020), On the other hand, only the precipitation method is not enough to remove Sr completely. Therefore, the combination to use precipitation and adsorption was discussed in this study. A variety of adsorbents have been developed including bentonite (El-Maghrabi et al., 2021), metal-oxygen/sulfide (Weerasekara et al., 2013), nanocarbon materials, graphene oxide (Huo et al., 2021), zeolite (Yang et al., 2021), titanic acid/phosphoric acid/antimonite (Kasap et al., 2012; Zhang et al., 2016; Jiao et al., 2021), etc. Biochar is prepared from natural biomass or agricultural waste that has widely concerned due to its unique advantages such as naturally renewable, biodegradable, easily adjustable surface structure, and environmentally friendly (Choudhary et al., 2020; Imran et al., 2021), making it an ideal adsorbent for removal Sr-90. In this study, the modified pomelo peel was used because of the large amount of pomelo production in China and the contents of rich functional groups, large specific surface area, and mesoporous structure. Total grapefruit and pomelo production in China in 2020–21 is estimated at 4.95 million metric tons, a negligible increase from the previous year and the slight increase is mainly driven by production of grapefruit hybrids in Guangxi and Yunnan (Citrus industry news, 2020). Though Naringin and naringenin in pomelo peers have strong antihyperglycaemic properties, large amout of pomelo peels were discarded and the utilization of pomelo peel wastes were necessary.

However, the application of biochar for the removal of high concentrations of strontium in the aqueous phase suffers from poor selectivity and low removal efficiency. On the other hand, in recent years, nanobubbles, as an emerging Frontier technology, have been widely favored by researchers for their unique advantages of long-term stability, high zeta potential, high surface-area-to-volume ratio, and generation of free radicals upon collapse (Zhou et al., 2021). To address the aforementioned limitations, this paper creatively adopts a combination of modified activated carbon adsorption and nanobubbles precipitation to achieve efficient removal of strontium from seawater because the precipitation reaction with nanobubble gas is rapid. The modified biochar materials were prepared by alkali impregnation and strong acid oxidation. Advanced analytical techniques including SEM-EDS, BET, EA, TG-DSC, XRD, FTIR, and XPS were employed to obtain important parameters of the adsorbent and reveal the interaction mechanism between the adsorbent and strontium. The static adsorption and desorption behaviors were evaluated by batch experiments. In this study, CO_2_ nanobubble co-precipitation was used to remove the majority of Sr(II) from artificial seawater, and the remaining Sr(II) in the solution was adsorbed by as-prepared biochar adsorbent.

## 2 Experimental

### 2.1 Materials and Reagents

The pomelo peel used in the experiment was purchased in Nanning City, Guangxi Province.

The simulated seawater concentrations for this study: 10,402 ppm Na(I), 1,275 ppm Mg(II), 390 ppm K(I), 405 ppm Ca(II) and 50 ppm Sr(II) (Shahzad et al., 2021).

All the chemicals (NaOH, HNO_3_, H_2_SO_4_, NaCl, KCl, MgCl_2_, CaCl_2_, SrCl_2_⋅6H_2_O) were purchased from Aladdin Biochemical Technology Co., Ltd (Shanghai) and were of analytical grade.

### 2.2 Preparation of Pomelo Peel Biochar

The pomelo peel was cut into strips and cleaned several times with ultrapure water to remove dust and impurities on the surface, and then dried in an oven at 80°C for 24 h. The dried pomelo peel was first crushed and then sieved through a 0.25 mm sieve. The broken pomelo peel was pyrolyzed in a muffle furnace at 500°C for 2 h with a heating rate of 10°C/min under continuous nitrogen purging. Finally, the biochar obtained by air cooling to room temperature was labeled as PMB.

### 2.3 Oxidation Modification Treatment

Different mass ratios of biochar/sodium hydroxide were mixed, and then 50 ml of ultrapure water was added and stirred continuously for 3 h to mix thoroughly with sodium hydroxide, then filtered and dried in an oven at 80°C until the water was completely removed. The dried impregnated biochar was pyrolyzed for 2 h at 500°C with a heating rate of 10°C/min in a muffle furnace with N_2_ airflow. The resulting sample was washed with 8 M nitric acid for 3 h to neutralize the remaining sodium hydroxide and oxidize the impregnated biochar, and the biochar was washed until the pH was stable with ultrapure water (about pH 4.5–5). Then it was dried for 6 h at 80°C in an oven, and stored in a dry box in a sealed bag for later use. The obtained samples were labeled as PMBNn (n = 1, 2, 3, 4, 5), representing that the ratios of biochar/NaOH were 1:1, 1: 2, 1: 3, 1: 4, and 1: 5, respectively.

### 2.4 The Nanobubble and Normal Bubble Production Method

In this study, CO_2_ (99.5% CO_2_) was injected into the 50 nm UFB generator (KITZ Engineering service Co, Ltd.) to produce nanobubbles at a flow rate of 5 L/min. Normal bubbles were not generated using the UFB generator, but by passing a plastic tube directly into the artificial seawater.

### 2.5 Characterization

The surface morphology of the adsorbent was obtained by SEM (HITACHI, SU8200, Japan) combined with EDX (Phenom ProX, Holland). The structural parameters, including surface area and pore size distribution, of the adsorbent were measured by BET (TriStar, II 3020, United States). Adsorbent element composition was acquired by an automatic EA (Elemantary, Vario EL cube, Germany). TG-DSC (NETZSCH, STA 449F3, Germany) was used to investigate the thermal stability of original and modified biochar under oxygen atmosphere. FTIR (Thermo, Nicolet IS 10, United States) was used to determine the surface chemical functional groups of the original biochar and modified biochar before and after adsorption. XPS (Thermo, K-Alpha+, United States, C1s: 284.6 eV) was adopted to analyze the chemical state of the elements.

### 2.6 Batch Adsorption Experiments

A 1.0 g (Sr(II))/L stock solution was prepared. The adsorption behaviors of as-prepared adsorbents towards Sr(II) were studied. The effects of pH (1–9), *m*/*v* (0.25–3.5 g/L), contact time (0–180 min), and initial Sr(II) concentration (40–100 ppm) were investigated. The biochar was added to a glass bottle with the Sr-containing solution and then shaken at a fixed speed (140 rpm). The suspension was separated with a 0.45 μm syringe filter, and the metal ion concentrations in the aqueous phase were measured by ICP-AES (ICPS-7510, Shimadzu, Japan) and AAS (SHIMADZU, AA-7000, Japan). Several key parameters, including adsorption capacity (*Q*) and adsorption efficiency (E), were calculated. The details were described in the supplementary information (SI).

The pH_PZC_ of the adsorbent is obtained by the pH drift method (Mironyuk et al., 2019). Add 20 ml of 0.1 M NaCl solution as an inert electrolyte into a 50 ml glass bottle with a lid, and adjust the initial pH from 1 to 9 with HCl and NaOH. After accurately measuring the initial pH value, 0.04 g of modified biochar was added to each Erlenmeyer flask, and the solution was stirred for 3 h to reach equilibrium. After 3 h, the equilibrium pH of the solution was measured and plotted against the initial pH, and the zero point charge was calculated and determined.

## 3 Results and Discussion

### 3.1 Optimization of the Preparation Process

Sodium hydroxide modification can increase surface area and oxygen-containing functional groups (i.e., -OH, -COOH) (Wang and Wang, 2019). Considering the poor physical and chemical properties of unmodified biochar, five different concentrations of NaOH solutions were selected to modify the surface morphology and internal structure of biochar. SEM images of biochar before and after modification (Supplementary Figure S1A–F). The surface of PMB has irregular cavities, voids, and pores, which may be due to water loss and the release of volatiles from the biomass matrix (Uçar et al., 2014) (Supplementary Figure S1A). The modified biochar (Supplementary Figure S1B–E) possesses a well developed porous structure due to the interaction between NaOH and carbon (Zhang et al., 2019). However, with the concentration of NaOH further increasing, the porous structure of biochar can be damaged (Choudhary et al., 2020) (Supplementary Figure S1F). The adsorption performance of as-prepared adsorbents towards Sr(II) was studied.

**FIGURE 1:**
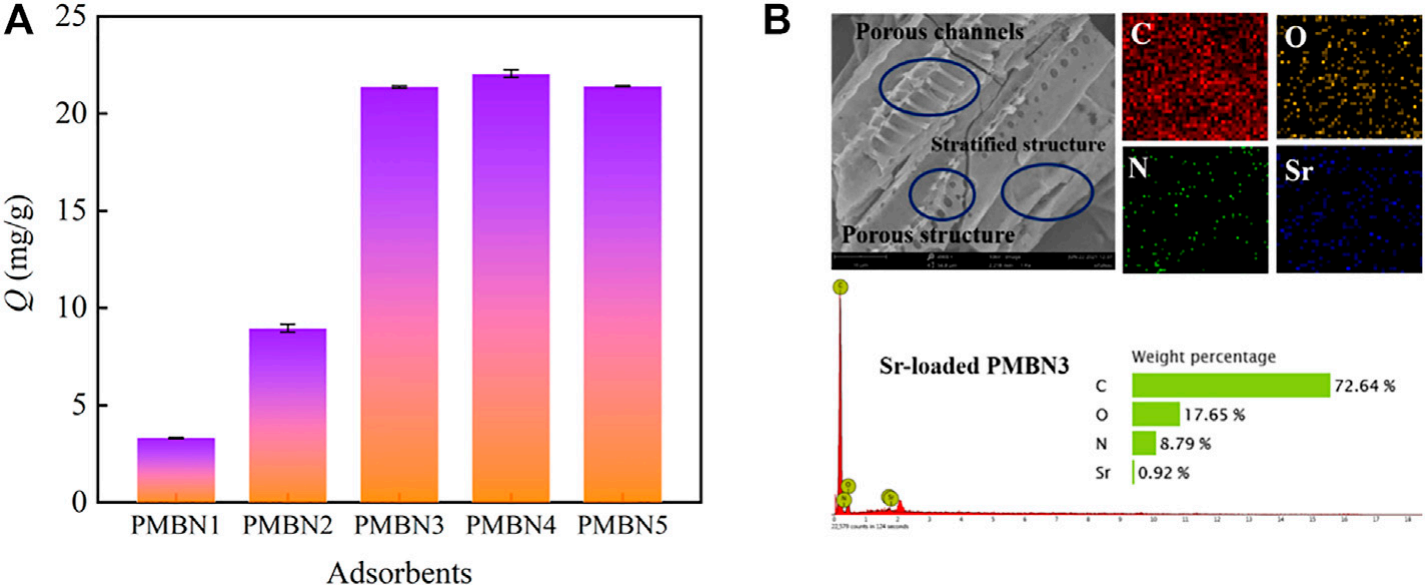
**(A)** Effect of impregnant ratio on Sr(II) adsorption (*C*_o_: 50 ppm, *m*/*v*: 2 g/L, *T*: 298 K, *t*: 3 h, *r*: 120 rpm), **(B)** SEM-EDS picture of PMBN3.

As shown in Figure 1A, the as-prepared adsorbents exhibit favorable adsorption behavior toward Sr(II) with the NaOH concentration increase. PMBN3 was selected for further adsorption experiments. The modified biochar has obvious pore channels and stratified structure. The results of the EDS of adsorbed PMBN3 showed that Sr was successfully adsorbed on the biochar surface (Figure 1B).

The elemental composition and main structural parameters of the biochar are summarized in Table 1. As the proportion of sodium hydroxide increases, the carbon content decreases, and the oxygen and hydrogen content increase. Compared with the unmodified biochar, the H/C ratio of the modified biochar remains unchanged, but the O/C and [(O+ N)/C] ratio increase. The molar H/C ratio of charcoal is commonly used to describe the degree of carbonization of biochar (Zhang et al., 2019). The H/C ratio of all biochars is less than 0.5, with lower H/C ratio indicating strong carbonation and high aromaticity (Li et al., 2021). The molar O/C ratio of the charcoal partly reflects its surface hydrophilicity, with the unmodified biochar having an O/C ratio of 0.19, indicating a low surface affinity for water, whereas the modified biochar has a substantially higher affinity for water, indicating a high content of polar functional groups (Chun et al., 2004). The polarity index [(O+ N)/C] rises with an increasing NaOH ratio, which indicates that the polar functional groups on the surface of the modified biochar have increased (Samsuri et al., 2013).

**TABLE 1:** The results of elemental analysis and main structural parameters of unmodified biochar and modified biochar.

Sample	Elemental composition analysis (wt%)				Atomic ratio			BET surface area (m^2^/g)	Average pore diameter (nm)	Pore volume (cm³/g)
	C [%]	H [%]	N [%]	O [%]	H/C	O/C	(O + N)/C			
PMB	75.31	2.98	1.69	14.15	0.04	0.19	0.21	2.35	7.68	0
PMBN1	72.27	3.10	3.34	21.54	0.04	0.30	0.34	379.31	2.29	0.22
PMBN2	70.42	3.27	2.63	27.19	0.05	0.39	0.42	762.16	2.17	0.41
PMBN3	62.05	3.40	2.65	32.35	0.06	0.52	0.56	1819.22	2.17	1.02
PMBN4	64.49	3.63	2.38	32.92	0.06	0.51	0.55	1911.44	2.21	1.06
PMBN5	66.14	3.72	2.68	41.30	0.06	0.62	0.66	1823.02	2.25	0.99

### 3.2 Characterization of PMBN3 Adsorbent

**FIGURE 2:**
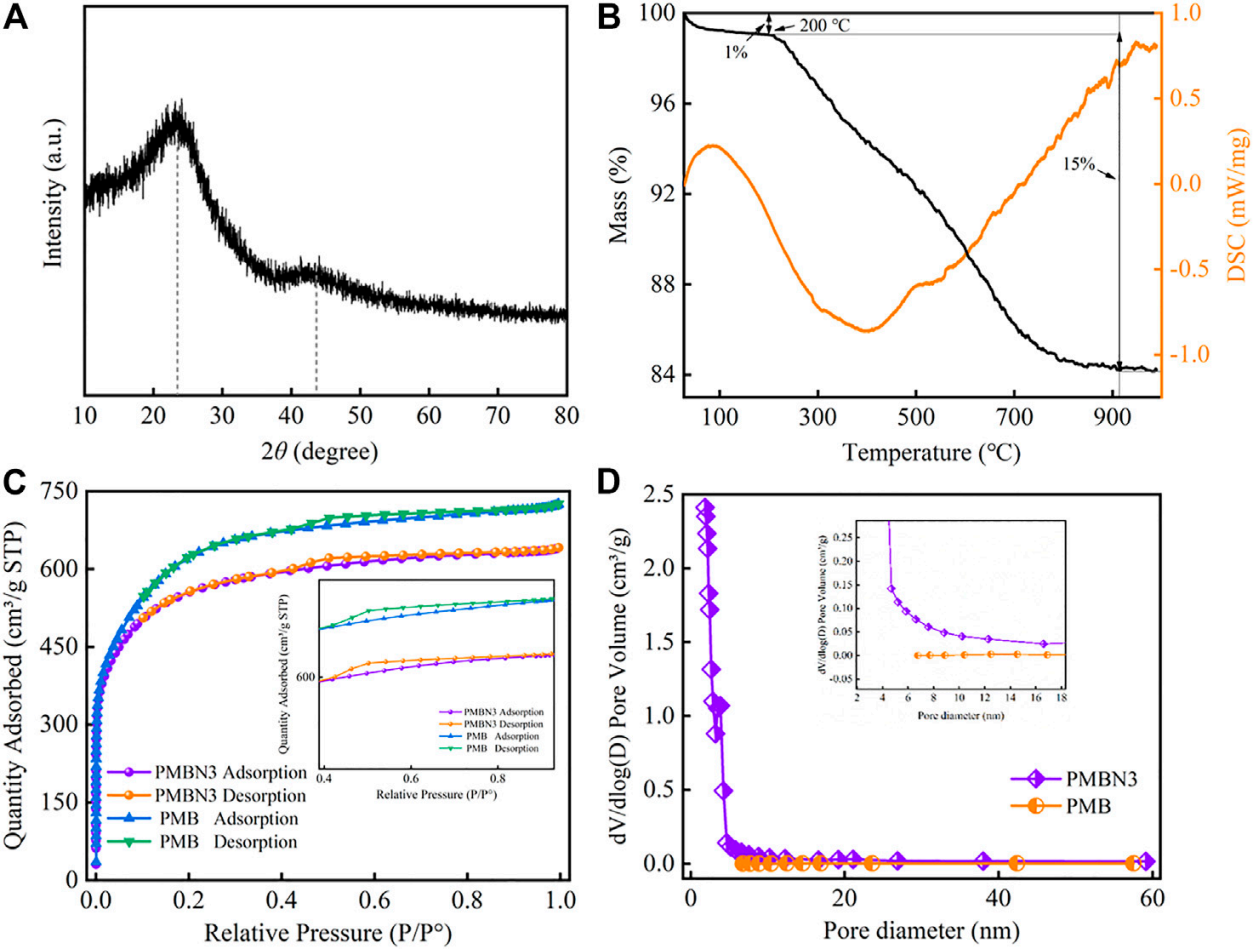
**(A)** The XRD patterns and **(B)** TG-DSC curves of PMB and PMBN3 biochar, **(C)** N_2_ adsorption-desorption isotherm, and **(D)** pore diameter distribution of PMB and PMBN3 biochar.

The XRD pattern of PMBN3 is illustrated in Figure 2A. Two diffraction peaks at 2θ = 23^o^ and 43^o^ were observed, which corresponded to the (002) and (100) peaks of the disordered graphite (Xu et al., 2014), indicating that PMBN3 possesses a microcrystalline and turbohydrostatic graphite structure (Prasannamedha et al., 2021). No other obvious peaks were observed except for these two peaks. The increase in background signal between 2θ = 10°–20° may be due to the abundant micropores and mesopores on the surface of biochar. These porous structures will cause the X-ray beam to scatter, resulting in a significant increase in the background XRD signal (Younis et al., 2020).

Figure 2B shows the TG-DSC results of PMBN3. At temperatures ranging from 50 to 200°C, PMBN3 loses a small amount of mass due to the evaporation of residual water and the decomposition of surface volatile organic compounds (Wu et al., 2017). With the temperature further increasing to 900°C, the TG curves show that the mass loss of PMBN3 adsorbent is about 15%, which is mainly attributed to the thermal decomposition of the organic components (Yang et al., 2007). As the temperature continues to increase the mass is essentially constant, which is attributed to the aromatic carbon contained in the structure enhancing the stability of the carbon structure (Li et al., 2021). According to DSC results, the endothermic peak at 88°C contributed to the evaporation of residual water and the loss of volatile organic compounds. A broad exothermic peak was observed at about 400°C, which results from the decomposition of hemicellulose, cellulose, and lignin (Boumanchar et al., 2017).

The N_2_ adsorption-desorption isotherms of PMB and PMBN3 are shown in Figure 2C, the curves of PMB and PMBN3 have a narrow hysteresis loop that fits well with type-IV isotherm curves, indicating PMBN3 is a kind of mesoporous material (Zhang et al., 2021). According to Table 1, compared with PMB, PMBN3 attained a high BET surface area (1819.22 m^2^/g), large pore volume, and small average pore diameter, which were beneficial for capturing Sr(II). The pore diameter distribution of PMB and PMBN3 were acquired (Figure 2D), combining with the results of Table 1, the average pore diameter is 2.2 nm, indicating the mesoporous structure of PMBN3 adsorbent.

### 3.3 Batch Adsorption Experiments

#### 3.3.1 pH and Zero-point Charge Study

**FIGURE 3:**
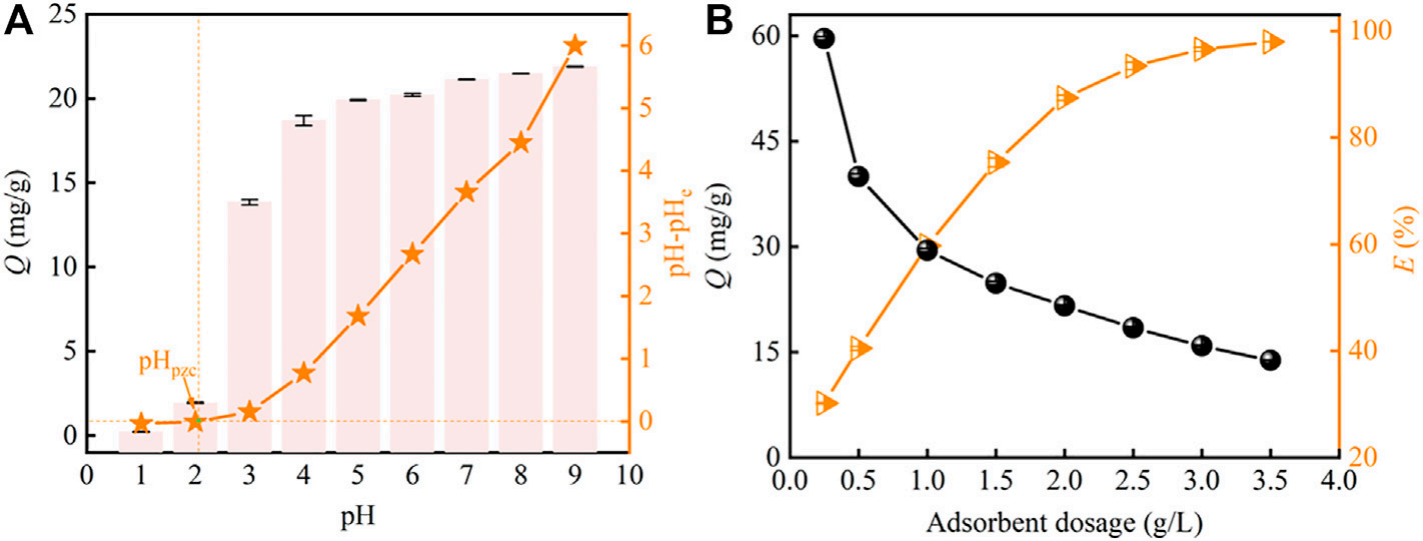
**(A)** Effect of pH on Sr(II) adsorption (*C*_o_: 50 ppm, *m*/*v*: 2 g/L, *T*: 298 K, *t*: 3 h, *r*: 120 rpm). **(B)** effect of adsorbent dosage on Sr(II) adsorption (*C*_o_: 50 ppm, pH: 8, *T*: 298 K, *t*: 3 h, *r*: 120 rpm).

The effect of pH on strontium adsorption by PMBN3 adsorbent is displayed in Figure 3A. The adsorption of Sr(II) by the PMBN3 rises rapidly with the increase of pH and finally tends to the adsorption equilibrium. Because deprotonation of functional groups (hydroxyl and carboxyl) and ion-exchange capacity are suppressed at pH 1-2, PMBN3 exhibits poor adsorption towards Sr(II). During the adsorption process, H^+^/H_3_O^+^ competes with Sr(II) and is preferentially adsorbed (Hassan et al., 2020). The adsorption of PMBN3 towards Sr (II) increased significantly due to the enhanced deprotonation of functional groups when the pH increased from 2 to 6. As the pH further increased from 6 to 9, the positive charge density on the adsorbent surface decreased, the electrostatic repulsive force weakened, and the concentration of free hydroxyl groups in solution increased, resulting in a slight increase in adsorption under alkaline conditions (Dan et al., 2020). The experimental results above were verified by the relationship between pH and pHzpc. When pH < pHpzc, the surface of the adsorbent is positively charged, which is not conducive to the adsorption of Sr(II). When pH > pHpzc, due to the ionization of acidic functional groups, the negatively charged biochar becomes an electron donor and the positively charged Sr(II) is easily adsorbed on the surface of the biochar through ion exchange. When pH > 9, Sr(II) is easily hydrolyzed to form hydroxide complex precipitates (Sr(OH)_2_) (Younis et al., 2020). In this study, the surface charge of PMBN3 is also investigated by measuring the zeta potential at different pH (Supplementary Figure S2). The results are in general agreement with those obtained by the charge drift method. Therefore, pH 9 was chosen as the upper limit in this study to prevent Sr(II) precipitation. Besides, the actual seawater pH is generally between 8.0 and 8.5, so the further experimental pH is 8.

#### 3.3.2 Effect of PMBN3 Dosage

The effect of PMBN3 dosage on Sr(II) adsorption was evaluated (Figure 3B). When the different PMBN3 dosages from 0.3 to 3.5 g were added into a 1 L Sr-containing solution, the adsorption efficiency of Sr(II) increased first and then reached equilibrium, conversely, the adsorption capacity gradually decreases. The increase in the adsorbent dosage provides more adsorption sites. From Figure 3B, the adsorbent dosage continues to increase, the adsorption site is not saturated but the adsorption capacity increases slowly, which may be due to the reduction of total active surface area and electrostatic interaction caused by the aggregation of adsorbent particles (Suliman et al., 2020).

#### 3.3.3 Kinetic Study

**FIGURE 4:**
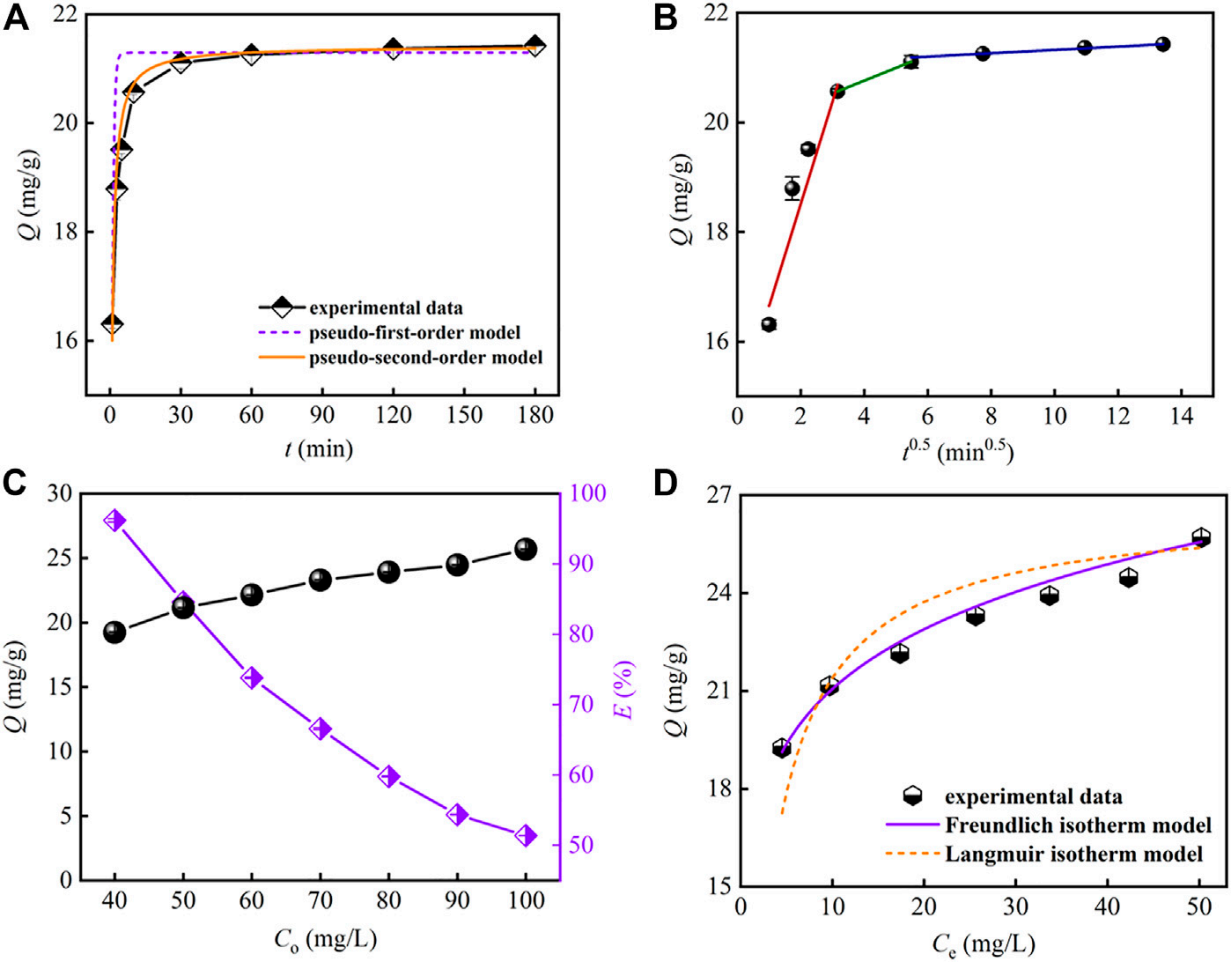
**(A)** Adsorption kinetics, **(B)** intra-particle diffusion, **(C)** effect of initial concentration on Sr(II) adsorption (*m*/*v*: 2 g/L, pH: 8, *T*: 298 K, *t*: 3 h, *r*: 120 rpm), **(D)** adsorption isotherm.

Figure 4A depicts the effect of contact time on Sr(II) removal with other parameters kept constant. From the figure, the adsorption capacity of PMBN3 towards Sr(II) increases sharply and then reaches equilibrium within 60 min with the adsorption capacity of about 21 mg/g. To better understand the adsorption style of PMBN3 towards Sr(II), three typical adsorption kinetic models (i.e., pseudo-first-order, pseudo-second-order, and intra-particle diffusion model; more details were described in SI) were adopted to analyze experimental data. According to Figure 4A and Table 2, the fitting results of the Pseudo-second-order model possess a higher correlation coefficient (*R* ^2^) and better consistency between *Q*_e_ and *Q*_e,exp_, which indicates that the adsorption process of PMBN3 towards Sr(II) was chemisorption. This indicates that the chemisorption rate is the limiting step (more details were described in Section 3.4 adsorption mechanism). Since the adsorption process is controlled not only by external mass transfer but also by pore diffusion, the internal diffusion model was further investigated.

**TABLE 2:** Kinetic parameters of PMBN3 adsorption towards Sr(II).

Kinetic models	Parameters	—
Pseudo-first-order	*q*_e_ (mg⋅g^−1^)	21.29
	*k*_1_ (min^−1^)	1.44
	*R* ^2^	0.76
Pseudo-second-order	*K*_2_ (g⋅mg⋅min^−1^)	0.34
	*q*_e_ (mg⋅g^−1^)	21.41
	*R* ^2^	0.98
Intraparticle diffusion model	*K*_IPD,1_	1.87
	*C*_1_	14.78
	*R*_1_^2^	0.94
	*K*_IPD,2_	0.23
	*C*_2_	19.83
	*R*_2_^2^	1
	*K*_IPD,2_	0.03
	*C*_2_	21.02
	*R*_2_^2^	0.97

The fitting results of the intra-particle diffusion model are shown in Figure 4B. The adsorption process mainly contained three diffusion stages, which suggests multiple adsorption mechanisms. The diffusion rate constant of the first stage is relatively large, which indicates that the adsorption is faster. Sr(II) is rapidly transferred from the solution to the adsorbent’s outer surface. In the second stage, Sr(II) diffuses into the pores, and in the third stage, Sr(II) slowly adsorbs on the inner surface of PMBN3 until the adsorption equilibrium is reached. None of the three straight lines cross through the origin, indicating that intra-particle diffusion may not be the only adsorption limiting mechanism (Cazetta et al., 2011).

#### 3.3.4 Isotherm Study

The relationship between the equilibrium adsorption capacity of PMBN3 towards Sr(II) and the initial Sr-containing solution concentration was investigated. According to Figure 4C, the *Q*_e,exp_ increases as the concentration of Sr-containing solution increases. To further evaluate the adsorption performance of PMBN3 towards Sr(II), several typical adsorption isotherm models (i.e., Langmuir, Freundlich, Temkin, and Dubinin-Radushkevich models, more details are described in SI) were used to analyze the experimental data (Figure 4D; Table 3). Adsorption isotherms are also used to describe the surface properties and affinity of the adsorbent (Dan et al., 2020). According to Table 3, the *R* ^2^ of the Freundlich model is much closer to 1 compared with that of other models, indicating that the adsorption process of PMBN3 towards Sr(II) matched well with the Freundlich model. Those findings imply the adsorption process is a non-homogeneous multilayer process (Huo et al., 2021). Besides, the high *R* ^2^ of the Temkin model suggests that the binding energy between adsorbent and metal ions was uniformly distributed (Al-Ghouti and Da’ana., 2020) (Supplementary Figure S3). Furthermore, the adsorption performance of PMBN3 towards Sr(II) was verified by the D-R model. The R2 > 0.96 means an excellent linear relationship between ε2 and lnQe (Supplementary Figure S4). The mean free energy is 9.70 kJ/mol (*E* > 8 kJ/mol), indicating the adsorption process is dominated by chemisorption (Zhang et al., 2021).

**TABLE 3:** Isotherm parameters of PMBN3 adsorption towards Sr(II).

Isotherm models	Parameters	—
Langmuir isotherm	*K*_L_ (L⋅mg^−1^)	0.41
	*q*_m_ (mg⋅g-1)	26.63
	*R* ^2^	0.89
Freundlich isotherm	*N*	8.31
	*K*_F_ (mg^1-n^⋅L^n^/g)	15.96
	*R* ^2^	0.98
D-R isotherm	*β* (mol^2^⋅J^−2^)	5.31 × 10^–9^
	*E* (kJ⋅mol^−1^)	9.703
	*R* ^2^	0.96
Temkin isotherm	*K*_t_ (L/mg)	5.34
	*Β*	2.76
	*R* ^2^	0.97

#### 3.3.5 Adsorption Thermodynamic Study

**FIGURE 5:**
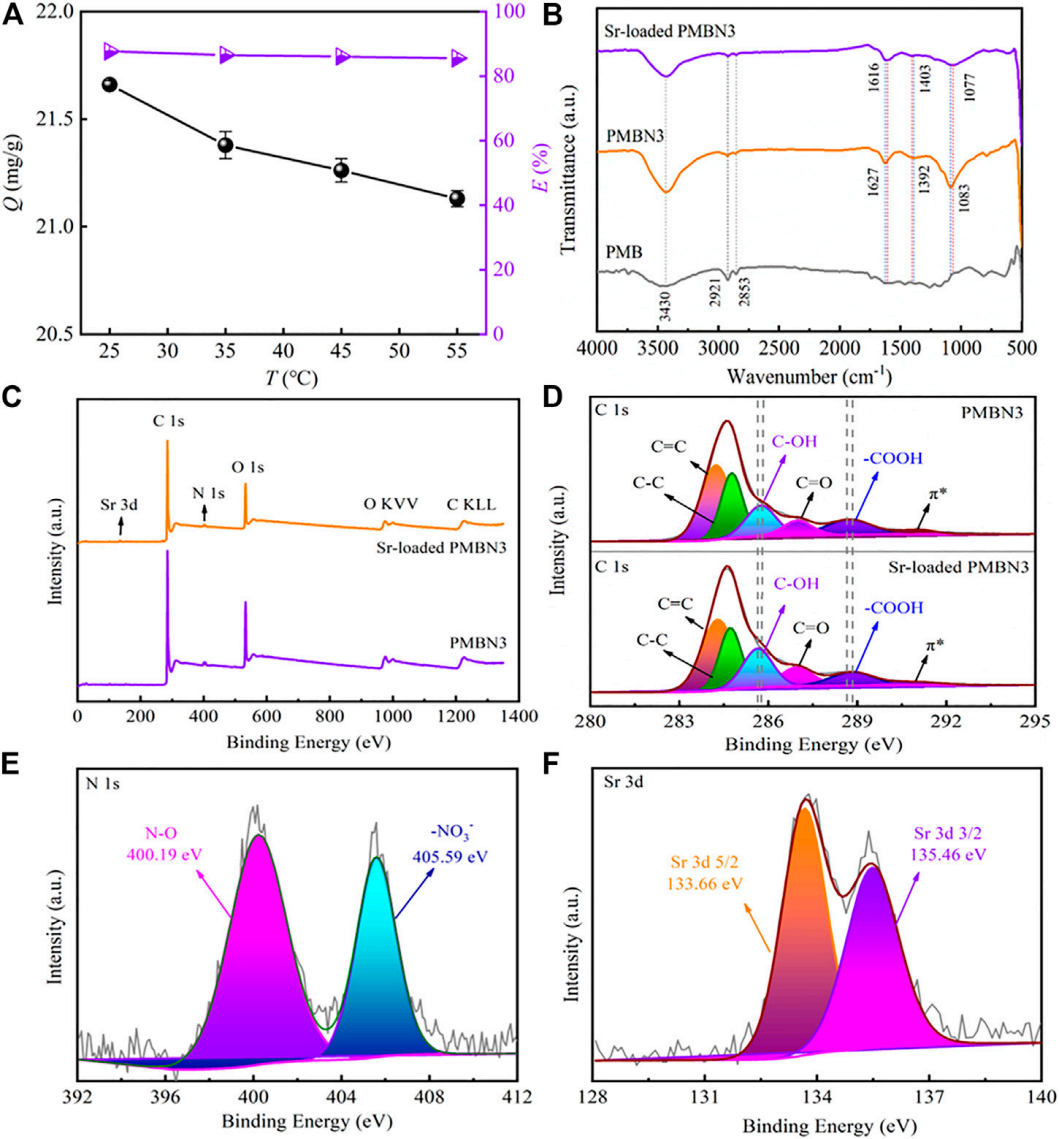
**(A)** Effect of temperature on Sr(II) adsorption (*C*_o_: 50 ppm*, m*/*v*: 2 g/L, pH: 8, *t*: 3 h, *r*: 120 rpm), **(B)** FTIR spectra of PMB, PMBN3 and Sr-loaded PMBN3, **(C)** XPS survey scans of PMBN3 and Sr-loaded PMBN3, **(D)** XPS spectra of C 1s, **(E)** XPS spectra of N 1s, **(F)** XPS spectra of Sr 3 days.

Figure 5A shows the effect of temperature on Sr(II) adsorption. According to Figure 5A, the adsorption capacity decreases with increasing temperature, indicating that adsorption of PMBN3 towards Sr(II) is an exothermic process (Hafizi et al., 2011), but temperature has little effect on adsorption in terms of removal rate. The thermodynamic parameters are calculated and presented in Table 4 (calculation formula and further details are described in SI). The negative value of *ΔH* indicates that the adsorption of Sr(II) by PMBN3 is an exothermic process, which is consistent with the figure. *ΔG* < 0, indicating that the adsorption is feasible and spontaneous. The lower the temperature, the more negative the *ΔG* value, indicating that the lower the temperature, the more favorable the adsorption (Zazycki et al., 2018). A negative value of *ΔS* indicates that the randomness between the solid-liquid interface is reduced (Tang et al., 2019).

**TABLE 4:** Thermodynamics parameters of Sr(II) adsorption on PMBN3.

Temperature (K)	Thermodynamic parameters		
	∆G (kJ⋅mol^−1^)	∆H (kJ⋅mol^−1^)	∆S (J⋅mol^−1^⋅K^−1^) (J/mol K)
	(kJ/mol)	(kJ/mol)	
298.00	−20.27	−5.14	50.73
308.00	−20.68		
318.00	−21.25		
328.00	−21.80		

### 3.4 Adsorption Mechanism

The FTIR spectra of PMB, PMBN3, and Sr-loaded PMBN3 are shown in Figure 5B. The O-H stretching vibration of the adsorbed water causes the broad peak at 3,430 cm^−1^. (Chen et al., 2019). The asymmetric stretching of the aliphatic -CH and -CH2 in the carbonyl group is responsible for the peaks at 2,921 and 2,853 cm^−1^, indicating that biochar contains cellulose and hemicellulose (Choudhary et al., 2020). The peaks around 1,600 cm-1 correspond to C=C, C=O, and C=N stretching vibrations, which represent the carboxyl (-COOH), carbonyl group (-C=O) and imine bond (-C=N) (Sahin et al., 2017; Shang et al., 2020). The band near the wavenumber of 1,400 cm^−1^ is related to the -COO group (Dong et al., 2017). After adsorption, the peaks at 1,627 and 1,403 cm-1 were shifted to 1,616 and 1,392 cm^−1^, respectively, suggesting that the metal ion had been chelated to the carboxyl group (Hu et al., 2020). The peak at 1,077 cm^−1^ may be associated with the C-O of the carboxylic acid group, the C-OH bonding of the alcohols, and the C-C bending vibrations (Samsuri, et al., 2013; Abdelhafez and Li., 2016), which shifted from 1,086 cm^−1^ to 1,077 cm^−1^ after adsorption. Compared with the unmodified biochar, the modified biochar has a high oxygen content and a significant number of oxygen-containing functional groups, which remains consistent with the results of the elemental analysis. The oxygen in the carboxyl or carbonyl group and the hydroxyl group operated as strong Lewis bases due to the existence of non-bonded electron pairs, forming coordination bonds with the Sr (II) ions as Lewis acids during the adsorption process (Rao et al., 2009). After NaOH impregnation, small amounts of sodium ions form COO-Na^+^ with -COOH which is deprotonated under alkaline conditions, and the new ions formed can facilitate ion exchange adsorption of Sr (II) ions from aqueous solutions (Younis et al., 2020).

To further investigate the compositional changes and the adsorption mechanism during the adsorption process of PMBN3, XPS spectroscopy was used to analyze the property changes of the main element content (C, N and O) before and after adsorption. The presence of O and N may be due to the incomplete carbonization of carbohydrates in the biomass and the doping of N and the washing of HNO_3_ during the carbonization process (Li et al., 2016). Figure 5C shows the XPS patterns of modified biochar PMBN3 before and after adsorption. The peak of Sr3d can be observed in the pattern of Sr-loaded PMBN3, which indicates that strontium was successfully adsorbed. The patterns of C1s, N1s, and Sr3d are shown in Figures 5D–F. The C1s in biochar are divided into five peaks corresponding to the hybridized carbon atoms (C=C, C-C), hydroxyl group (C-OH), carbonyl group (C=O), carboxyl group (-COOH), and π-π* transition in aromatic hydrocarbons. The binding energies of those groups shift from 284.23 to 284.29 eV, 284.77 to 284.72 eV, 285.75 to 285.64 eV, 286.99 to 286.95 eV, 288.73–288.83 eV, and 291.06 to 291.00 eV, respectively (Huang et al., 2017; Hu et al., 2021; Li et al., 2021). The large binding energy changes indicate a strong affinity between the metal ions and the oxygen-containing functional groups, suggesting that the latter are participating in the adsorption reaction (Zhang et al., 2021).

**FIGURE 6:**
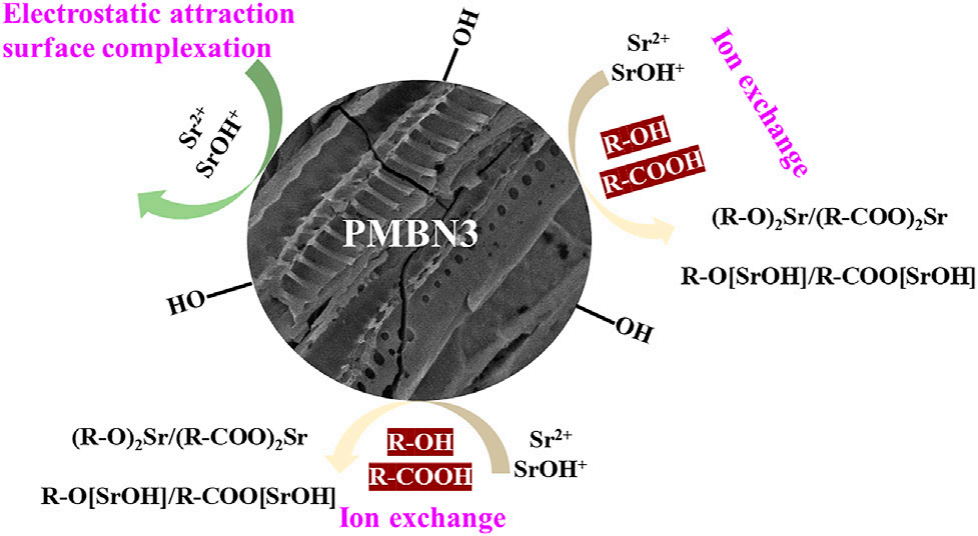
Adsorption mechanism of Sr(II) onto PMBN3.

Based on the results of EA, FTIR, and XPS, the adsorption mechanism between PMBN3 and Sr was revealed (Figure 6). The adsorption process involves the following: ⅰ) the interaction of a deprotonated oxygen-containing functional group on the PMBN3 adsorbent surface with Sr(II) (Huo et al., 2021); ⅱ) the weak van der Waals forces between Sr(II) and PMBN3 adsorbent (Shin et al., 2021b).

### 3.5 Desorption Performance and Reusability of PMBN3

**FIGURE 7:**
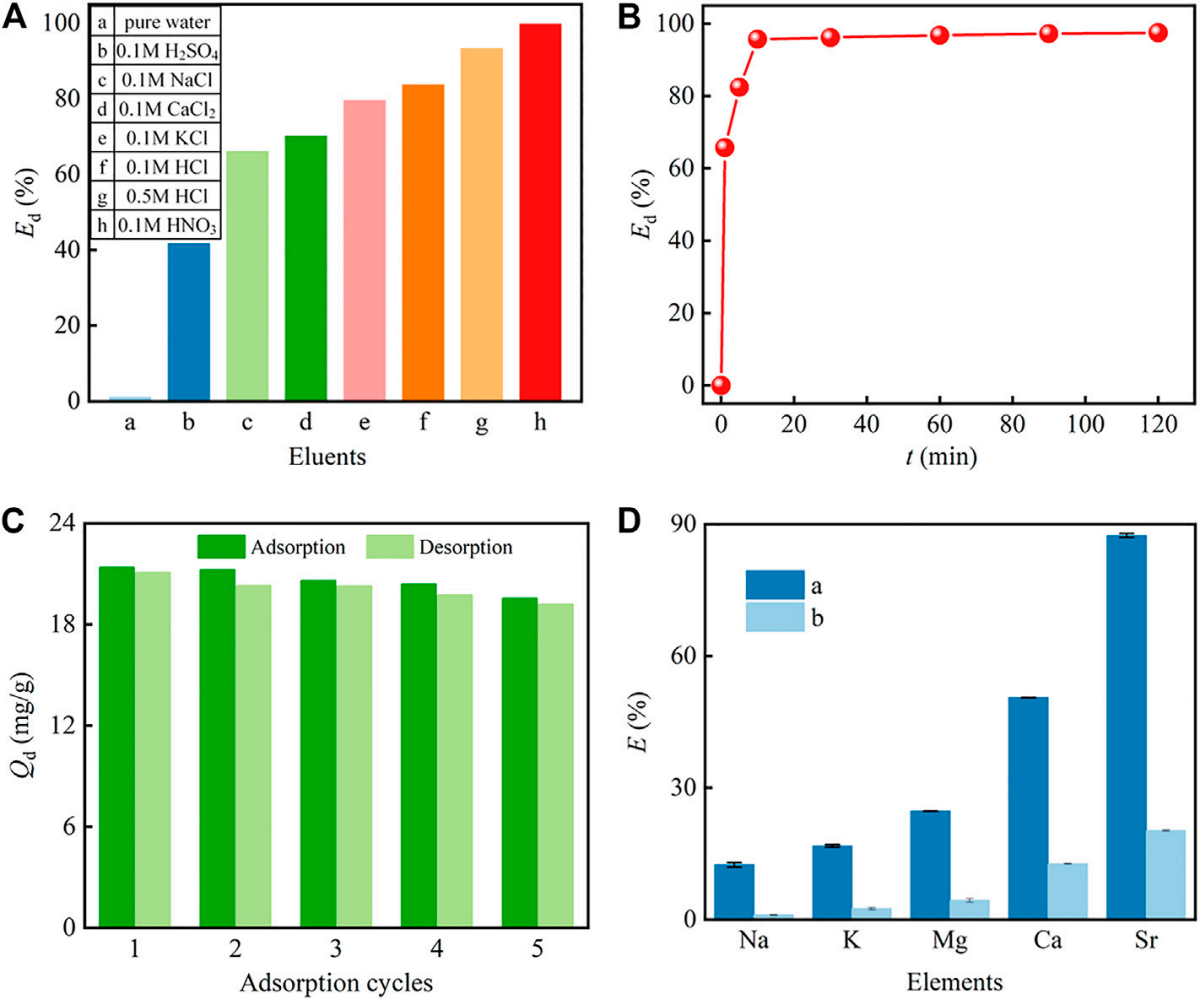
**(A)** Desorption efficiency of Sr(II) by various eluents, **(B)** kinetis study of the desorption process (Co: 50 ppm, medium: 0.1 M HNO_3_ solution, *m*/*v*: 2 g/L, *T*: 298 K, *r*: 120 rpm), **(C)** the reusability of PMBN3 (*C*_o_: 50 ppm, *m*/*v*: 2 g/L, *T*: 298 K, *t*: 1 h, *r*:120 rpm), **(D)** single-component and competitive adsorption (*C*_M_: 50 ppm).

Both the desorption performance and the reusability of the PMBN3 adsorbent are studied. The important parameters include the desorption amount *Q*_d_ and the desorption efficiency E_d_, which are calculated by Eq. S12) and (13), respectively. According to Figure 7A, different eluents are chosen to desorb Sr-loaded PMBN3 and exhibit great differences. The adsorbed Sr(II) can be effectively desorbed by 0.1 M HNO_3_ with the *E*_d_ over 99% and the desorption equilibrium can be obtained within 10 min (Figure 7B). Furthermore, 0.1 M HNO_3_ is employed to investigate the reusability of the PMBN3 adsorbent. From Figure 7C, PMBN3 shows excellent reusability, the adsorption capacity is only slightly reduced after five adsorption-desorption cycles, which implies that the PMBN3 has great potential in practical application.

### 3.6 Single-Component and Competitive Adsorption

The multicomponent environment of actual seawater may cause competitive adsorption between interfering ions and Sr(II). The adsorption performance of PMBN3 towards Sr(II) in both single component aqueous phase and artificial seawater was investigated. From Figure 7D, PMBN3 exhibits excellent adsorption performance towards Sr(II) with an adsorption rate of about 90% and poor or weak adsorption towards other metal elements in single component aqueous phase (*C*_o_ = 50 ppm).

Competitive adsorption is carried out by mixing different metal solutions at the same concentration (50 ppm) and the same process as for the single component adsorption (Figure 7D). The results show that PMBN3 still has the highest removal rate for strontium, but the removal rate is significantly lower compared to the single component solution. The figure shows the effects of Na(I), K(I), Mg(II), and Ca(II) on Sr(II), respectively. The inhibition trend of these ions on Sr(II) is in order: Ca(II)>Mg (II)> K(I)> Na(I). The alkaline earth metals Ca(II) and Mg(II) inhibit the adsorption of Sr(II) because the same main group elements have similar chemical properties and ionic radii. The greater effect of Ca(II) on Sr(II) removal by PMBN3 than Mg(II) may be owing to the fact that the hydration ion radius of Sr(II) is almost the same as that of Ca(II), but both are smaller than that of Mg(II) (Zhang and Liu., 2020).

### 3.7 Sr(II) Precipitation by Blowing CO_2_ Nanobubble With Neutralization

For efficient removal of radioactive Sr(II) from contaminated seawater, CO_2_ nanobubbles are blown in to precipitate Sr(II) for environmental remediation. Configure a solution with a high concentration of competing ions to artificial seawater, and the following reactions will occur when CO_2_ enters the solution (Erdemoğlu and Canbazoglu., 1998):

$$CO_{2}+H_{2}O\overset{\;}{\Leftrightarrow }H_{2}CO_{3}$$ (1)

$$H_{2}CO_{3}\overset{\;}{\Leftrightarrow }H^{+}+HCO_{3}^{-}$$ (2)

$$K_{2}=\frac{\left[H^{+}\right]\left[HCO_{3}^{-}\right]}{\left[H_{2}CO_{3}\right]}=4.30\times 10^{-7}$$ (3)

$$HCO_{3}^{-}\overset{\;}{\Leftrightarrow }H^{+}+CO_{3}^{2-}$$ (4)

$$K_{4}=\frac{\left[H^{+}\right]\left[CO_{3}^{2-}\right]}{\left[HCO_{3}^{-}\right]}=5.61\times 10^{-11}$$ (5)

$$CO_{3}^{2-}+M^{2+}\overset{\;}{\Leftrightarrow }MCO_{3}$$ (6)

At this time, the pH changed from 8 to 3.72, and the pH of the seawater solution was then adjusted using 10 M NaOH to keep it at 9. The particle size and potential of the nanobubbles were measured and the results showed 3,621 nm and −1.59 mV, respectively. The larger average particle size is due to the presence of a small number of large bubbles, but most of them are nanobubbles (Supplementary Figure S5).

**FIGURE 8:**
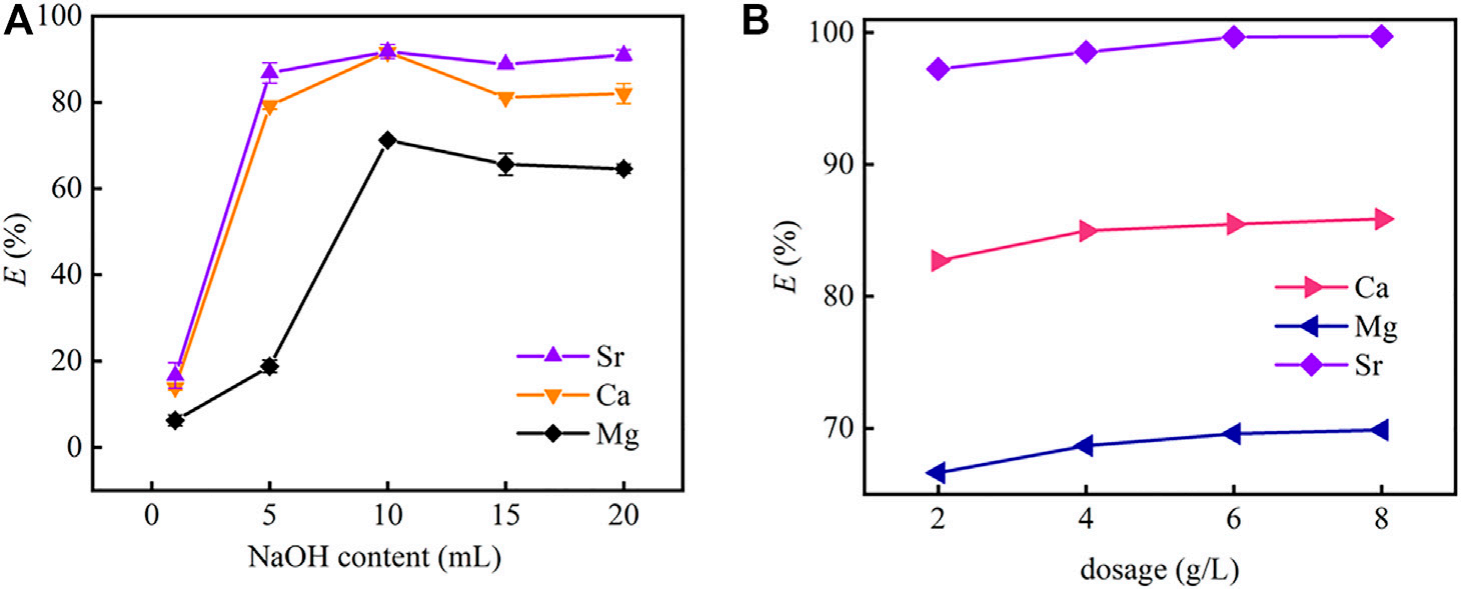
**(A)** The effect of the concentration of NaOH on the precipitation of strontium by carbon dioxide nanobubbles with no adsorbent, **(B)** effect of PMBN3 adsorbent dosage on Sr(II) adsorption after precipitation (pH: 9, *T*: 298 K, *t*: 3 h, *r*: 120 rpm).

Figure 8A shows the effect of the content of NaOH on the precipitation of strontium by CO_2_ nanobubbles. The amount of precipitated Sr(II) (mainly SrCO_3_) increases with NaOH content first, and reaches its highest at 10 ml with a removal rate of over 95%, then decreases slightly with NaOH content further increasing. From Section 3.3, PMBN3 is unable to completely remove Sr(II) from seawater. To improve the removal rate of Sr(II) from seawater, chemisorption and nanobubble precipitation methods were employed and combined. According to Figure 7B, the removal rates of CO_2_ nanobubbles for Mg, Ca, and Sr(II) were 64.6, 82.0, and 95.9%, respectively. The as-prepared PMBN3 was applied to the adsorption of residual metal ions from the aqueous phase after precipitation, and the removal rates reached 69.9% (for Mg), 85.9% (for Ca) and 99.7% (for Sr) with increasing the PMBN3 (the detailed concentration of ions involved before and after CO_2_ precipitation and adsorption in the experiment is described in SI). The precipitation was analyzed by XRD. The XRD patterns of the precipitate was dominated by peaks of MgCO_3_.3H_2_O,characteristic peaks appeared at 2θ = 23.0°, 29.5°, 36.0°,47.6°, confirming that the precipitate contained CaCO_3_. No strontium carbonate precipitate was detected because the strontium concentration was too low (Figure 6).

\The results of normal bubbles and nanobubbles were basically the same (Supplementary Figure S7A,B **)**, but comparing the effects of ordinary bubbles and nanobubbles on the precipitation time, nanobubbles reacted in water and rapidly consumed sodium hydroxide to produce carbonate precipitation, and the time was drastically reduced **(** Supplementary Figure S8 **)**.

## 4 Conclusion

In this study, at first 95.9% of Sr(II) ion was removed by precipitation with CO_2_ nanobubbles from 50 ppm Sr(II) included artificial seawater. Next, the adsorption was carried out. Here, more detailed adsorption characteristics were investigated. A novel teak peel modified biochar (PMBN3) was prepared using rapid pyrolysis and oxidative modification to remove Sr(II) from artificial seawater. The characterization results of the as-prepared PMBN3 reveal the properties of the rich functional groups, large specific surface area, and mesoporous structure. Through batch adsorption and desorption experiments, rapid adsorption kinetics (<1 h for equilibrium), superior reusability of PMBN3 were obtained, and the adsorption of PMBN3 towards Sr(II) matched well with pseudo-second-order, intra-particle diffusion kinetic models and Freundlich, Temkin and Dubinin-Radushkevich isotherm models, which indicated it is multilayer chemisorption. XPS and FTIR results revealed that the ion exchange mechanism and ectrostatic attraction surface complexation in Sr(II) adsorption. Furthermore, considering the deficiency that biochar cannot completely remove high concentration of Sr(II) from artificial seawater, blowing CO_2_ nanobubbles into Sr-containing solution to precipitate the interfering metal ions, and followed by the adsorption of PMBN3 towards residual metal ions with the removal rate of Sr(II) over 99%, which confirmed that the combination of chemisorption and nanobubble precipitation techniques can achieve efficient removal of Sr(II). As the used NaOH amount is large for 50ppm Sr(II) precipitation by using CO_2_ nanobubble, this system is useful to the limited high concentrated radioactive strontium water. In summary, the combination of traditional adsorption and emerging nano-bubble technology for strontium removal is not only promising but also provides a new reference for future environmental remediation.

## Data Availability

The original contributions presented in the study are included in the article/Supplementary Material, further inquiries can be directed to the corresponding author.
