# Supplementary material for: Strontium Ion Removal From Artificial Seawater Using a Combination of Adsorption With Biochar and Precipitation by Blowing CO2 Nanobubble With Neutralization
Source: Front Bioeng Biotechnol. 2022 Feb 10;10:819407. doi: 10.3389/fbioe.2022.819407 (PMC8866730; doi:10.3389/fbioe.2022.819407)
Supplement: Supplementary file 1 [file DataSheet1.docx]

**Supplementary information**

**3. Results and Discussion**

**3.1. Optimization of the preparation process**


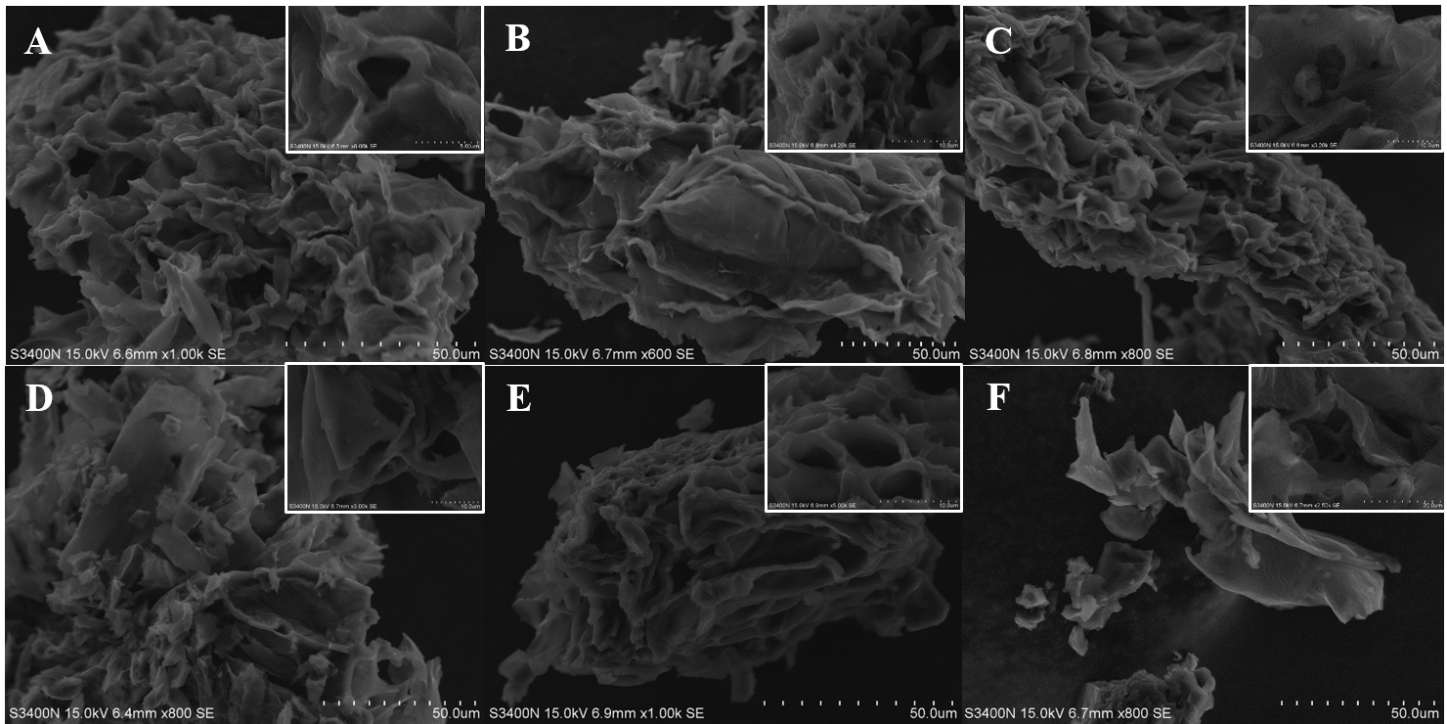


**Fig. S 1** SEM images of biochar with different ratios of impregnation ratios (a: Unmodified biochar; b: Biochar : NaOH =1:1; c: Biochar : NaOH =1:2; d: Biochar : NaOH =1:3; e: Biochar : NaOH =1:4;f: Biochar : NaOH =1:5).

**3.3.1. pH and zero-point charge study**

**
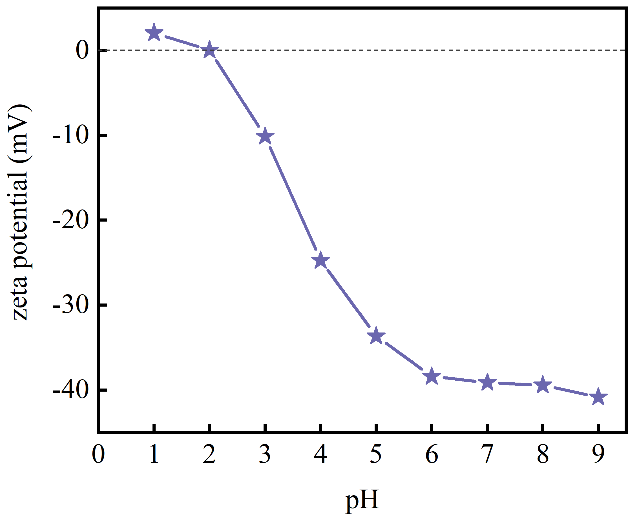
**

**Fig. S 2** Zeta potential of PMBN3 surface

The effect of zeta potential on Sr(II) adsorption by PMBN3 increases with increasing pH. The zero potential point of PMBN3 is near pH 2. At pH 1.0-2.0, the positive value of zeta potential indicates the protonation of the adsorbent surface, which is not favorable for Sr(II) adsorption. At pH 2.0-7.0, the negative value of zeta potential gradually decreases, corresponding to an increase in the negative charge on the sample surface, which is extremely favorable for Sr(II) adsorption.

**3.3.3. Kinetic study**

In this work, both pseudo-first-order kinetics model (Eq. S1)[1], pseudo-second-order kinetic model (Eq. S2)[1], and intra-particle diffusion model (Eq. S3)[2] were applied to analyze the experimental data.

| $\text{q}_{\text{t}}\text{=}\text{q}_{\text{e}}\left( \text{1-}\exp\left( \text{-}\text{k}_{\text{1}}\text{t} \right) \right)$ | S1 |
| --- | --- |
| $\text{q}_{\text{t}}\text{=}\frac{\text{q}_{\text{e}}^{\text{2}}\text{k}_{\text{2}}\text{t}}{\text{1+}\text{q}_{\text{e}}\text{k}_{\text{2}}\text{t}}$ | S2 |
| $\text{q}_{\text{t}}\text{=}\text{k}_{\text{p}}\text{t}^{\frac{\text{1}}{\text{2}}}\text{+C}$ | S3 |

Where *q*_e_ (mg·g^-1^) and *q*_t_ (mg·g^-1^) are the equilibrium adsorption capacity and adsorption capacity at time *t* (h) respectively; *k*_1_ (h^-1^), *k*_2_ (mg·g^-1^ h^-1^) and *k*p (mg·g^-1^·h^-1/2^) are the adsorption rate constant of pseudo-first-order, pseudo-second-order and intra-particle diffusion model respectively. *C* is a constant associated with the thickness of the boundary layer.

**3.3.4 Isotherm study**

Langmuir model[3] (Eq. S4) assumes the process is single layer adsorption that all the sites are evenly distributed and have the same adsorption ability. Freundlich model[3] (Eq. S5) assumes a heterogeneous adsorption surface with sites having different adsorption energies. The Temkin model[4] describes the interaction between the adsorbent and the adsorbate, and the correlation coefficient, indicates that the binding energy is uniformly distributed. The Dubinin-Radushkevich model[2] is widely used to predict physical/chemical adsorption and to estimate the average adsorption energy.

| $\text{q}_{\text{e}}\text{=}\frac{\text{q}_{\text{m}}\text{K}_{\text{L}}\text{C}_{\text{e}}}{\text{1+}\text{K}_{\text{L}}\text{C}_{\text{e}}}$ | S4 |
| --- | --- |
| $\text{q}_{\text{e}}\text{=}\text{K}_{\text{F}}\text{C}_{\text{e}}^{\frac{\text{1}}{\text{n}}}$ | S5 |
| $\text{q}_{\text{e}}\text{=}\frac{\text{RT}}{\text{b}_{\text{T}}}\text{In}\text{A}_{\text{T}}\text{C}_{\text{e}}$ | S6 |
| $\text{q}_{\text{e}}\text{=}\text{q}_{\text{D}}\text{exp} \left( \text{-}\beta_{\text{D}}\text{ε}_{\text{D}}^{\text{2}} \right)$ | S7 |

where *q*_e_ (mg·g^-1^) and *q*_m_ (mg·g^-1^) are equilibrium adsorption capacity and calculated saturation adsorption capacity; *C*_e_ (mmol·L^-1^) means equilibrium ions concentration; *K*_L_ (L·mg^-1^) and *K*_F_ (mg^1-n^·L^n^/g) are constants of Langmuir and Freundlich isotherm model; n means adsorption intensity. A_T_ and b_T_ are the constants. *q*_D_ (mg·g^-1^) calculated saturation adsorption capacity in the D-R model. *β*_D_ (mol^2^·J^-2^) is a constant related to adsorption energy. ε_D_ is the Polanyi potential (J·mol^-1^).

| $\text{ε}_{D}\text{=RT}\ln\left( \text{1+}\left( \frac{\text{1}}{\text{C}_{\text{e}}} \right) \right)$ | S8 |
| --- | --- |
| $\text{E= }\frac{\text{1}}{\sqrt{\text{2}\beta_{\text{D}}}}$ | S9 |

where R is the ideal gas constant (8.31447 J·mol^-1^K^-1^). The mean free energy *E* (J·mol^-1^) represents the energy change of Sr from solution to adsorbent. When the energy higher than 8 kJ mol^-1^, the process dominated by chemical adsorption, on the contrary, the process dominated by physical adsorption.





**Fig. S 3** Temkin isotherm model of PMBN3 towards Sr.





**Fig. S 4** D-R isotherm model of PMBN3 towards Sr.

**3.3.5. Adsorption thermodynamic study**

The thermodynamic parameters including enthalpy (ΔH, kJ⋅mol^−1^), entropy (ΔS, J⋅mol^−1^⋅K^−1^), and Gibbs free energy (ΔG, kJ⋅mol^−1^) were calculated using the following equations:

| $\text{In}\text{K}_{\text{d}}\text{=}\frac{\text{Δ}\text{S}}{\text{R}}\text{-}\frac{\text{Δ}\text{H}}{\text{R}}\frac{\text{1}}{\text{T}}$ | S10 |
| --- | --- |
| $\text{ΔG=ΔH-TΔS}$ | S11 |

where *K*_d_ is the distribution coefficient; *T* (K) is the adsorption temperature; *R* refers to the universal gas constant with the value of 8.314 J⋅mol^−1^⋅K^−1^.

## **3.5 Desorption performance and reusability of PMBN3**

The desorption method is the same as the adsorption method. The adsorbed biochar was added to a glass bottle with the different eluents and then shaken at a fixed speed (140 rpm). The desorption amount Q_d_ (mg·g^-1^), desorption efficiency E_d_ (%) were calculated using the following equations:

| $Q_{d}=C_{d}\times\frac{V}{m}$ | S12 |
| --- | --- |
| $E_{d}=\frac{Q_{d}}{Q}\times100\%$ | S13 |

Table S 2 Summary of Sr(II) adsorption capacity of different biosorbents

| Adsorbent | Equilibrium time | Reusability | Isotherm model | Adsorption capacity | References |
| --- | --- | --- | --- | --- | --- |
| graphene oxide‑coated biochar | 1h | 5 cycle | Langmuir | 4.09 mg/g | [5] |
| spent coffee waste biochars | 8h | — | Langmuir | 6.62mg/g | [6] |
| magnetic banana peels | 0.5h | 3 cycle | Langmuir | 23.827 mg/g | [7] |
| pomelo peel biochar | 1h | 5 cycle | Freundlich | — | This study |
| commercial activated carbon | 8h | — | Langmuir | 32.79mg/g | [8] |
| spent coffee grounds | 8h | — | Langmuir | 51.81 mg/g | [8] |
| rice straw-based  biochar | 12h | — | Langmuir | 77.44mg/g | [9] |

**3.7. Sr(II) precipitation by blowing CO_2_ nanobubble with neutralization**

**
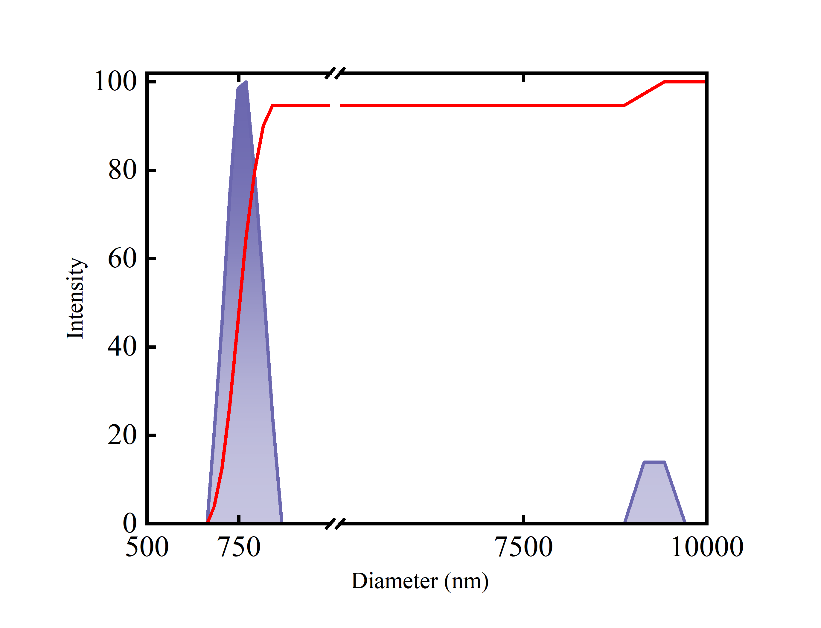
**

**Fig. S5** Size distribution of nanobubbles generated in artificial seawater

**Table S 2** The detailed concentration of ions involved before and after CO_2_ precipitation and adsorption

|  | Mg(ppm) | Ca(ppm) | Sr(ppm) |
| --- | --- | --- | --- |
| Before precipitation | 1275 | 405 | 50 |
| After precipitation | 451.55 | 72.82 | 2.04 |
| After adsorption | 384.29 | 57.27 | 0.14 |


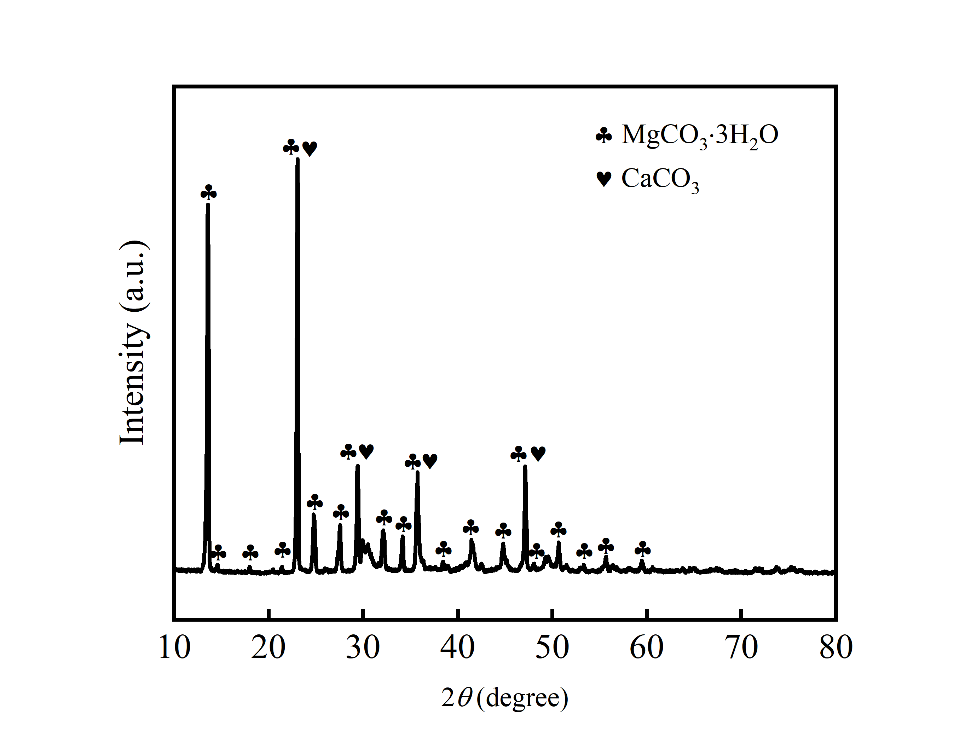


**Fig. S 8** XRD patterns of precipitation

The precipitation was analyzed by X-ray diffraction (XRD). In comparison with the standard data, the XRD pattern of the precipitate was dominated by peaks of MgCO_3_·3H_2_O with lattice constants a=7.71, b=5.37,c=12.12 (JCPDS70-1433), thus proving that the precipitate contains simple monoclinic structure of MgCO_3_·3H_2_O. characteristic peaks appeared at 2θ=23.0°, 29.5°, 36.0°, 47.6°, a= 4.98, b= 4.98, c=17.02 coincided with card JCPDS85-1108, confirming that the precipitate contained CaCO_3_. No strontium carbonate precipitate was detected because the strontium concentration was too low.

**
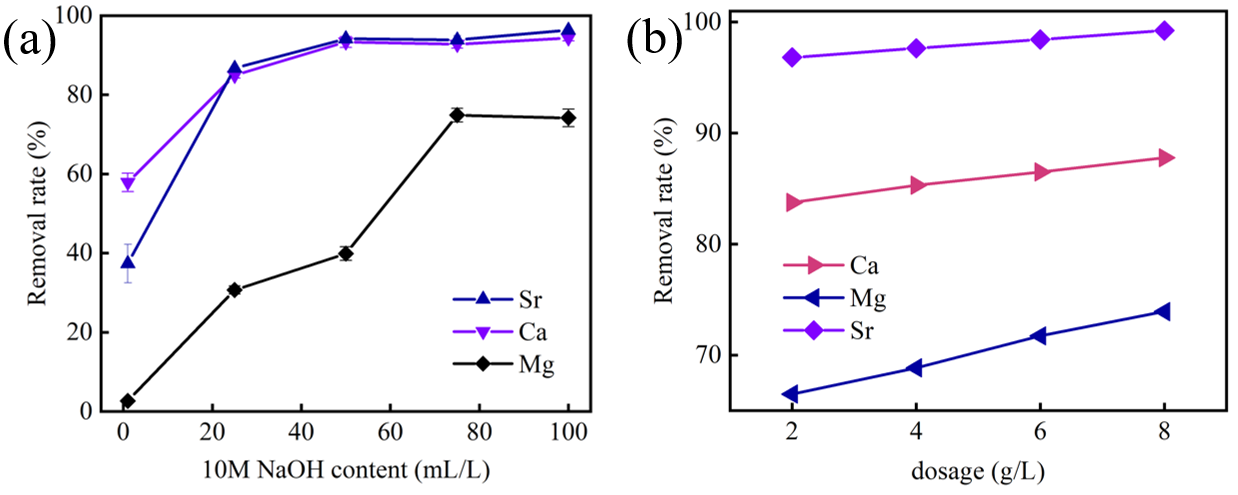
**

**Fig. S 6** (a) The effect of the concentration of NaOH on the precipitation of strontium by carbon dioxide normal bubbles with no adsorbent, (b) effect of PMBN3 adsorbent dosage on Sr(II) adsorption after precipitation (pH*:*9, *T*: 298 K, *t*:3h, *r*:120 rpm).


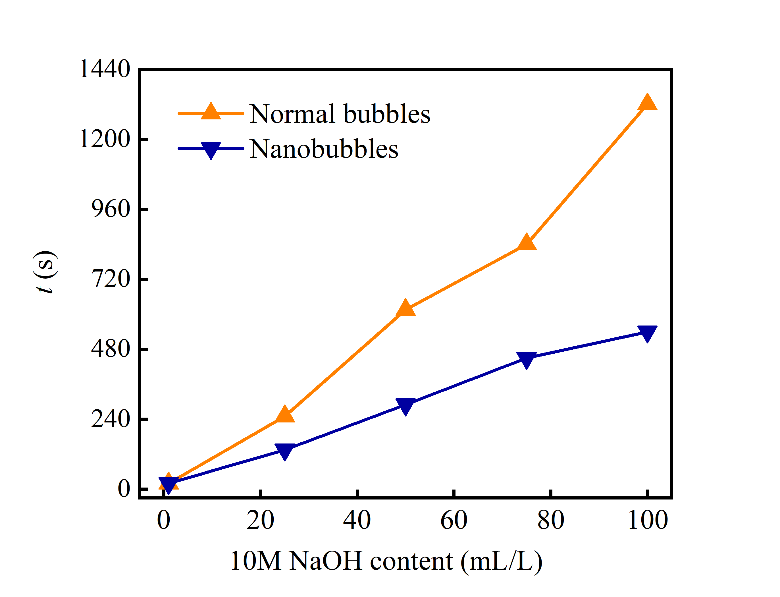


**Fig. S 7** Nanobubbles and normal-bubbles precipitation time.

**References**

[1] C. Liu, X. Yu, C. Ma, Y. Guo, T. Deng, Selective recovery of strontium from oilfield water by ion-imprinted alginate microspheres modified with thioglycollic acid, Chemical Engineering Journal, 410 (2021).

[2] S. Zhang, S. Ning, H. Liu, X. Wang, Y. Wei, X. Yin, Preparation of ion-exchange resin via in-situ polymerization for highly selective separation and continuous removal of palladium from electroplating wastewater, Separation and Purification Technology, 258 (2021).

[3] S.A. Younis, R.A. El-Salamony, Y.F. Tsang, K.-H. Kim, Use of rice straw-based biochar for batch sorption of barium/strontium from saline water: Protection against scale formation in petroleum/desalination industries, Journal of Cleaner Production, 250 (2020).

[4] P. Amesh, K.A. Venkatesan, A.S. Suneesh, U. Maheswari, Tuning the ion exchange behavior of cesium and strontium on sodium iron titanate, Separation and Purification Technology, 267 (2021).

[5] V. Chakraborty, P. Das, P.K. Roy, Synthesis and application of graphene oxide-coated biochar composite for treatment of strontium-containing solution, International Journal of Environmental Science and Technology, 18 (2020) 1953-1966.

[6] J. Shin, J. Kwak, Y.G. Lee, S. Kim, C. Son, K.H. Cho, S.H. Lee, Y. Park, X. Ren, K. Chon, Changes in adsorption mechanisms of radioactive barium, cobalt, and strontium ions using spent coffee waste biochars via alkaline chemical activation: Enrichment effects of O-containing functional groups, Environ Res, 199 (2021) 111346.

[7] J.W. Choi, S. Cho, S.J. Choi, Ecofriendly, selective removal of radioactive strontium ions in aqueous solutions using magnetic banana peels, Sci Total Environ, 778 (2021) 146327.

[8] J. Shin, S.H. Lee, S. Kim, D. Ochir, Y. Park, J. Kim, Y.G. Lee, K. Chon, Effects of physicochemical properties of biochar derived from spent coffee grounds and commercial activated carbon on adsorption behavior and mechanisms of strontium ions (Sr(2+)), Environ Sci Pollut Res Int, 28 (2021) 40623-40632.

[9] B.S. Reddy, A.K. Maurya, E.S. V, P.L. Narayana, M.H. Reddy, A. Baazeem, K.K. Cho, N.S. Reddy, Prediction of batch sorption of barium and strontium from saline water, Environ Res, 197 (2021) 111107.
